# Influence of Etching Protocols on the Bonding Stability of Universal Adhesives to Dentin

**DOI:** 10.3390/polym18121516

**Published:** 2026-06-18

**Authors:** Mehtap Kaba, Güneş Bulut Eyüboğlu, Muhammet Karadas

**Affiliations:** 1 Department of Restorative Dentistry, Faculty of Dentistry, Recep Tayyip Erdogan University, 53100 Rize, Turkey; mehtap.kaba@erdogan.edu.tr; 2 Department of Restorative Dentistry, Faculty of Dentistry, Karadeniz Technical University, 61080 Trabzon, Turkey; gunesbulut@ktu.edu.tr

**Keywords:** universal adhesive, bonding stability, degree of conversion, aging

## Abstract

This study aimed to assess the effect of etching protocols on the bonding performance of three universal adhesives to dentin under different aging conditions. Specimens were obtained from human molars and randomly allocated to 6 groups (*n* = 10) according to adhesive type (Clearfil S3 Bond Universal (CBU), Clearfil Universal Bond Quick (CUBQ), and Zipbond Universal (ZBU)) and application strategy (self-etch or etch-and-rinse). Each adhesive was applied according to the manufacturer’s instructions for the respective strategy, followed by composite resin build-up. Resin–dentin beams were prepared and subjected to three aging conditions: storage in distilled water at 37 °C for 24 h, 10,000 thermocycles, or pH cycling. Microtensile bond strength was measured, and the degree of conversion was evaluated using Fourier-transform infrared spectroscopy. Data were evaluated using analysis of variance and Weibull statistics (α = 0.05). Both adhesive type and application protocol significantly influenced bond strength (*p* < 0.05), whereas aging conditions had no significant effect (*p* > 0.05). At 24 h, ZBU in the etch-and-rinse strategy showed the highest bond strength and significantly outperformed CUBQ. After thermocycling and pH cycling, no significant differences were found among adhesives. All adhesives demonstrated higher reliability when used in the self-etch mode. Weibull analysis indicated that ZBU in the self-etch mode had the lowest probability of failure, with fractures occurring at higher stress levels. CUBQ showed the lowest degree of conversion.

## 1. Introduction

With the increasing use of composite resins in restorative dentistry, achieving durable adhesion between dental tissues and restorative materials has become a major clinical objective. Adhesive systems are designed to establish effective bonding to enamel and dentin while minimizing technique sensitivity, reducing marginal leakage, and preventing secondary caries [1]. In recent years, new-generation adhesive systems, referred to as “universal” or “multi-mode” adhesives, have been introduced. These systems allow the use of either etch-and-rinse (ER) or self-etch (SE) strategies [2].

In the ER strategy, phosphoric acid is applied to remove the smear layer, creating micromechanical retention sites on enamel and completely demineralizing dentin to a depth of several micrometers. This process exposes dentinal tubules and generates a collagen matrix devoid of hydroxyapatite, thereby facilitating resin infiltration and micromechanical bonding. The hybrid layer formed, along with resin tags within enamel and dentin, constitutes the fundamental mechanism of adhesion [3,4]. Although regarded as the gold standard for enamel bonding, the ER technique requires strict adherence to clinical protocols to achieve predictable outcomes. In contrast, SE adhesives integrate the conditioning and priming processes through the action of acidic functional monomers, eliminating the need for separate etching and rinsing procedures. This milder interaction with dental tissues creates a demineralized layer of only about 1 μm and preserves a portion of the hydroxyapatite surrounding collagen fibrils, which may enhance interfacial stability [3]. Although bond strength to enamel is generally lower with this approach, adhesion to dentin is supported by both micromechanical interlocking and chemical interactions with residual hydroxyapatite [5,6].

The question of which application mode yields the highest bonding efficiency on dentin remains a subject of ongoing debate in the literature [2]. Both bonding strategies present distinct advantages and limitations with respect to immediate performance as well as long-term bonding stability after aging [7]. The long-term performance of adhesive restorations depends on hybrid layer stability. Although degradation can occur with all bonding approaches, universal adhesives applied in the ER mode may be more susceptible due to their hydrophilic nature and incomplete resin infiltration into the demineralized collagen matrix. These factors may increase water uptake, accelerate interfacial degradation, and ultimately reduce bond durability [8,9].

The incorporation of acidic functional monomers, particularly 10-methacryloyloxydecyl dihydrogen phosphate (10-MDP), represents a defining characteristic that distinguishes universal adhesives from conventional single-step SE systems [10]. 10-MDP has been demonstrated to interact with hydroxyapatite via two principal adhesion mechanisms: (1) the formation of stable ionic bonds with calcium, resulting in a nanolayered structure composed of 10-MDP–Ca salts at the adhesive–hydroxyapatite interface [11,12,13], and (2) a relatively higher etching potential compared with other acidic functional monomers [14]. This hydrophobic nanolayering is considered to enhance the long-term stability of adhesion to both dentin and enamel [15]. Nevertheless, the precise contribution of nanolayering to bond durability remains a matter of ongoing debate. Numerous studies have investigated the bonding efficacy to dental structures and the clinical performance of universal adhesives; however, inconsistent and sometimes conflicting results have been reported regarding their relative advantages [16,17,18].

Previous meta-analyses and systematic reviews suggest that multiple factors, including the type and concentration of functional monomers in adhesive systems, the pH of the adhesive, as well as application strategy and clinical situations, may significantly affect bonding results [5,19,20,21]. However, comparative data on the durability of universal adhesives with different compositions when applied using different etching strategies and subjected to various aging procedures remain limited. Therefore, the present study aimed to compare the dentin bond strength of three universal adhesives with different compositions when applied in both SE and ER modes before and after two aging procedures. The tested null hypotheses were as follows: (1) the type of universal adhesive would not affect dentin bond strength; (2) the bonding protocol would not affect dentin bond strength; and (3) the aging protocol would not influence dentin bond strength.

## 2. Materials and Methods

### 2.1. Tooth Selection and Preparation

This study utilized extracted human molars obtained for therapeutic purposes unrelated to this study, in accordance with approval from the institutional Ethics Committee (Approval No. 2025/20). Teeth exhibiting caries, restorations, or structural defects were excluded. Eligible specimens consisted exclusively of fully erupted and intact molars. For disinfection, the teeth were immersed in Chloramine-T (0.5%) solution for seven days and subsequently maintained in distilled water, with storage not exceeding one month prior to specimen preparation. The teeth were embedded in acrylic resin using a silicone mold, extending 1–2 mm below the enamel-cementum junction. To expose the midcoronal dentin, each tooth was sectioned transversely using a low-speed cutting system under continuous water irrigation (Micracut 152, Metkon, Bursa, Turkiye). Consequently, all dentin surfaces were gently polished with 600-grit SiC paper for 30 s under standardized light pressure to produce a uniform smear layer [22]. The surfaces were scanned under a stereomicroscope (Stemi305, Göttingen, Germany) to verify the complete elimination of enamel.

### 2.2. Sample Size Calculation

In studies investigating bond strength using the micro-tensile bond strength test, the number of teeth per experimental group is commonly reported to range between 8 and 10 [23,24]. In line with these reports, ten teeth were allocated to each group. To confirm the adequacy of the sample size, a priori power analysis was conducted using G*Power software version 3.1.9.4 (Heinrich Heine University Düsseldorf, Düsseldorf, Germany)The analysis was performed based on F-tests and indicated that the selected sample size was sufficient for the study (effect size = 0.40; statistical power (1 − β) = 0.94).

### 2.3. Bonding Procedure

Sixty specimens were randomly allocated to 6 groups (*n* = 10) according to adhesive type (Clearfil S3 Bond Universal [CBU], Clearfil Universal Bond Quick [CUBQ], or Zipbond Universal [ZBU]), and bonding protocol (SE or ER). In the ER protocol, dentin surfaces were conditioned with phosphoric acid (35%, K-Etchant Syringe, Kuraray, Tokyo, Japan) for 15 s, washed with an air-water spray for 30 s, and gently dried. Adhesive agents were applied in accordance with the manufacturers’ instructions and subsequently polymerized for 10 s using a VALO LED device (1000 mW/cm^2^, Ultradent, South Jordan, UT, USA). Composite blocks of 6 mm were constructed by depositing 2 mm-thick layers of composite resin (3M ESPE, Filtek Z250 A2, St. Paul, MN, USA) on the bonded surfaces. Each layer was light-cured for 20 s with the light source. The restored samples were kept in distilled water at 37 °C for 24 h. All procedures were conducted by a single operator. The chemical composition and application procedure of the adhesives are presented in Table 1.

### 2.4. Micro-Tensile Bond Strength (Μtbs) Testing

Specimens were attached in a precision cutting device and sectioned with a diamond disc to obtain serial slices approximately 1 mm thick, initially in the buccolingual direction parallel to the long axis of the tooth. The blocks were subsequently rotated 90°, and sectioning was repeated in the mesiodistal direction. Resin–dentin beams were then produced by cutting the specimens along the cervical axis, perpendicular to the long axis of the tooth, yielding beams with an approximate cross-sectional area of 1 mm^2^ [25]. Beam thickness was verified using a digital micrometer with an accuracy of 0.02 mm. Specimens containing peripheral or residual enamel were excluded. The resin–dentin beams obtained from each tooth were randomly assigned in equal numbers to three aging conditions: 24 h of storage in distilled water, 10,000 thermal cycles, and pH cycling procedure, with a minimum of four beams included in each tooth. The bond strength assigned to each tooth was obtained by calculating the average bond strength of the resin–dentin beams derived from it. Pre-test failures were recorded as 0 MPa in the statistical analysis [26].

Thermal cycling was conducted in a PLC-controlled apparatus (Gökçeler Makine, Sivas, Turkey), with specimens alternately immersed in 5 °C and 55 °C water baths for a 30 s dwell time and 5 s transfer time, according to established protocols. This cycle was repeated 10,000 times, simulating a one-year aging effect [27]. For the pH-cycling model, resin–dentin beams from each subgroup were placed in separate containers and subjected to alternating demineralization and remineralization regimens. The beams were exposed to a 14-day pH-cycling protocol consisting of immersion in a demineralizing solution for 6 h at room temperature (2 mM KH_2_PO_4_, 2 mM Ca(NO_3_)_2_·4H_2_O, and 75 mM CH_3_COOH; pH adjusted to 4.3), followed by immersion in a remineralizing solution for 18 h (0.9 mM KH_2_PO_4_, 1.5 mM Ca(NO_3_)_2_·4H_2_O, and 130 mM KCl; pH adjusted to 7.0) [28,29]. Between cycles, the specimens were washed with distilled water, and both solutions were refreshed daily to maintain consistent experimental conditions.

At the end of the aging procedures, the resin–dentin beams were fixed to the grips of a microtensile testing unit (Bisco Inc., Schaumburg, IL, USA) equipped with a 500 N load cell using cyanoacrylate glue (Akfix 705, Istanbul, Türkiye) and subjected to tensile loading at a crosshead speed of 0.5 mm/min until failure. The peak load at the point of failure was recorded in Newtons. This value was then converted into bond strength (MPa) by dividing it by the bonded cross-sectional area. The fractured surfaces were then inspected under a stereomicroscope at 40× magnification, and the type of failure was classified as adhesive, dentin cohesive, composite cohesive, or mixed [30]. All experiments were conducted by one operator who was blinded to the group assignments.

### 2.5. Scanning Electron Microscopy (Sem) Assessment

To evaluate resin tag formation and the morphology of the adhesive surface, additional specimens were prepared following previously established protocols. Adhesive systems were applied using either the SE or ER protocols. The composite resin was incrementally layered over the bonded dentin surfaces and polymerized for 40 s. The restored specimens were maintained in distilled water at 37 °C for 24 h prior to further processing. For micromorphological evaluation, the tooth structures were dissolved by immersing the specimens in 6 N hydrochloric acid until complete dentin removal was achieved. Specimens were then washed with distilled water and exposed to 5% sodium hypochlorite for 10 min to eliminate residual organic components. After air drying, the specimens were sputter-coated with gold under vacuum and observed using SEM (JSM-6610, JEOL, Tokyo, Japan) at 1500× magnification to assess the adhesive surface characteristics and resin tag formation.

### 2.6. Degree of Conversion Assessment

The adhesives (*n* = 5) were analyzed for their degree of conversion (DC) using FTIR (Spectrum 100, PerkinElmer, Waltham, MA, USA) with an ATR accessory and diamond crystal. A thin layer of each adhesive was placed directly on the crystal to fully cover its surface. The spectra of the uncured adhesives were collected over a wavenumber range of 650–4000 cm^−1^, utilizing 32 scans at a resolution of 4 cm^−1^. After acquiring the baseline, a gentle stream of air was applied for 5 s to promote solvent evaporation. To reduce oxygen inhibition, the adhesive was covered with a transparent Mylar strip and subjected to light curing for 10 s, with the curing tip centrally positioned and in close proximity to the ATR crystal to ensure consistent light exposure. The post-curing spectra were recorded under the same conditions as the uncured measurements.

The ATR–FTIR spectra were meticulously processed and evaluated using OriginPro 2021 software (OriginLab Corp., Northampton, MA, USA). To ascertain the degree of conversion (DC), the ratio of the absorbance of the aliphatic C=C peak (~1638 cm^−1^) to the aromatic C=C peak (~1607 cm^−1^) was calculated both prior to and following polymerization, as previously documented [31]. The DC values were derived using the following formula:

$$DC\;\left(\%\right)=\left[\;1-\frac{\left(\frac{1638\;{cm}^{-1}}{1607\;{cm}^{-1}}\right)cured}{\left(\frac{1638{cm}^{-1}}{1607{cm}^{-1}}\right)uncured}\right]\times 100$$

### 2.7. Statistical Analysis

To minimize the risk of pseudo-replication arising from multiple beams obtained from the same tooth, the mean μTBS value of all beams from each tooth was used as the experimental unit for statistical analysis. Normality was evaluated using the Kolmogorov–Smirnov test, and variance homogeneity was assessed with Levene’s test. The μTBS data were analyzed through a three-way ANOVA, with adhesive type, application mode (SE or ER), and aging protocol as fixed factors, followed by Tukey’s post hoc test for multiple comparisons. The DC data were assessed using a one-way ANOVA with the Bonferroni test (*p* < 0.05). In addition, bond reliability and failure probability were examined using Weibull analysis. The Weibull shape (*m*) and scale (*σ*θ) parameters were determined using the maximum likelihood estimation method. The shape parameter reflects the reliability of the material, whereas the scale parameter represents the bond strength corresponding to a 63.2% probability of failure.

## 3. Results

### 3.1. Μtbs Analysis

**Figure 1:**
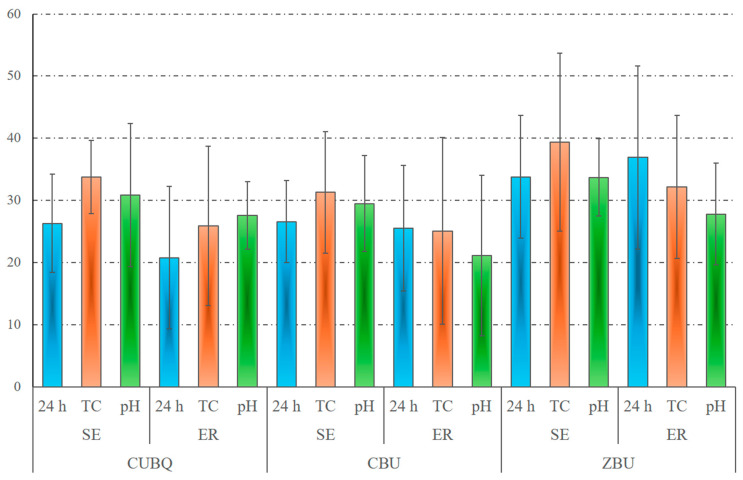
Mean microtensile bond strength values with standard deviation error bars for adhesives in different etching modes (self-etch (SE) and etch-and-rinse (ER)) after various aging protocols (24 h, thermocycling (TC), and pH cycling).

**Figure 2:**
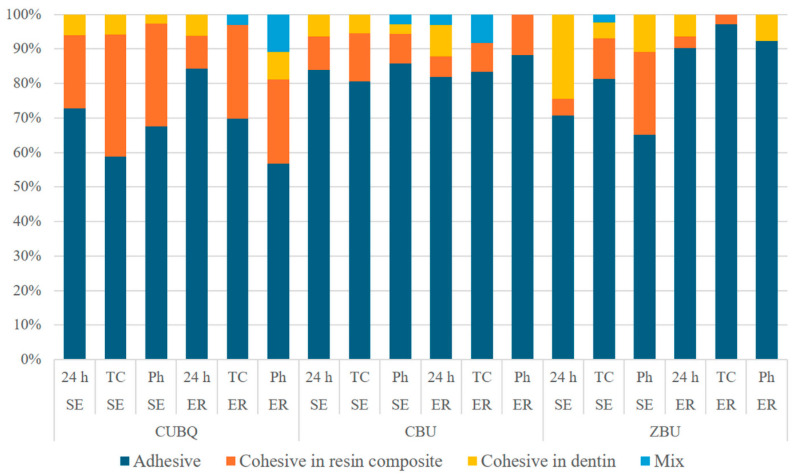
Stereomicroscopy failure analysis presenting the failure-mode distribution of adhesives in self-etch (SE) or etch-and-rinse (ER) protocols.

The mean μTBS values (±SD) are summarized in Table 2. Statistical analysis revealed that the adhesive agent (*p* = 0.001) and the application mode (*p* = 0.007) had a significant effect on bond strength. However, the aging protocols did not have a statistically significant effect on bond strength (*p* = 0.280). Furthermore, the interactions among all factors were found to be insignificant (*p* > 0.05). At 24 h, ZBU in the ER protocol exhibited the highest μTBS (Figure 1), showing a significant difference compared with CUBQ (*p* < 0.001). At 24 h, no significant differences were found among the remaining groups (*p* > 0.05). After thermocycling and ph-cycling aging, ZBU in the SE protocol exhibited the highest μTBS (Figure 1), but no significant differences were found between the groups (*p* > 0.05). Failure mode analysis revealed that adhesive failures were the predominant pattern across all experimental groups, irrespective of the adhesive system (CUBQ, CBU, and ZBU), bonding protocol (SE and ER), or testing time (24 h, TC, and pH level). Although minor variations were observed among the groups, cohesive failures in the resin composite or dentin and mixed failures remained considerably less frequent (Figure 2).

**Figure 3:**
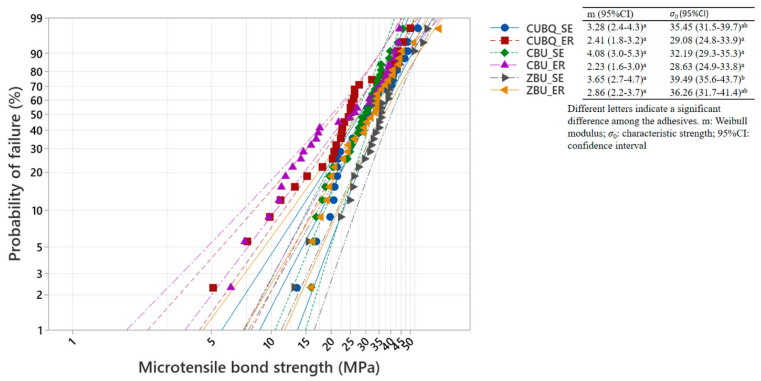
Weibull analysis of adhesive failure probability, regardless of testing time.

Figure 3 illustrates the Weibull reliability analysis and corresponding probability of failure for the tested adhesives, irrespective of testing time. Overall, adhesives applied in the SE protocol demonstrated greater reliability than those applied in the ER protocol, but no significant difference was found. ZBU in the SE protocol showed the lowest probability of failure among the adhesives, with failures occurring at higher stress levels during mechanical loading. In addition, all adhesives demonstrated a lower probability of failure in the SE protocol compared with the ER protocol, but no significant difference in failure probability was found between the two bonding protocols.

### 3.2. Sem Analysis

**Figure 4:**
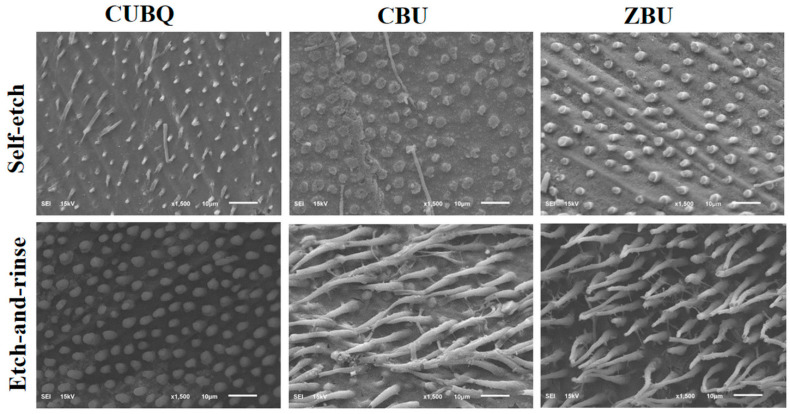
Representative SEM photomicrographs of adhesive surfaces. CUBQ: Clearfil Universal Bond Quick; CBU: Clearfil S3 Bond Universal; ZBU: Zipbond Universal.

Figure 4 shows SEM images of the adhesive layer formed on dentin surfaces using adhesives applied in SE and ER protocols. Morphological differences were observed between the adhesive layers formed with the two application protocols. In the SE application, thin resin tags were observed in the image of the CUBQ adhesive. In the SE images of ZBU and CBU adhesives, thicker resin tag formation was observed. After ER application, CBU and ZBU adhesives similarly showed longer and denser resin tag formation, while CBQ adhesive showed shorter and sparser resin tag formation.

### 3.3. Dc Analysis

The DC values (±SD) are shown in Table 3. Significant differences in DC were found among the adhesives (*p* = 0.012). CUBQ showed significantly lower DC compared to CBU (*p* = 0.029) and ZBU (*p* = 0.023). No significant difference was found between CBU and ZBU (*p* = 0.307).

## 4. Discussion

This study evaluated the bonding potential of universal adhesives in different application modes after different aging protocols. Statistical analysis revealed that both adhesive type and bonding protocol significantly affected dentin bond strength. Therefore, the first and second null hypotheses were rejected. However, the aging protocol did not affect the bond strength; thus, the third null hypothesis was accepted.

ZBU presented the highest bond strength in the SE or ER modes, but statistically similar performance to the other adhesives. Given the inherent variability of bond strength data, as observed in this study [32], Weibull analysis was employed to provide a more comprehensive assessment of adhesive reliability and failure probability [33]. While all adhesives exhibited comparable reliability within each bonding protocol (SE or ER), ZBU in the SE protocol demonstrated a notably lower probability of failure, suggesting improved resistance under higher stress levels. SEM images also revealed that ZBU showed better penetration into the dentin surface and shorter and thicker resin tags. In SE adhesive systems, bonding mainly occurs through chemical interactions between hydroxyapatite and functional monomers [3,34]. Although universal adhesives tested contain 10-MDP, the success of this interaction depends on sufficient monomer infiltration and the formation of a uniform and durable hybrid layer [35].

In the ER protocol, ZBU showed better performance, irrespective of the testing period (Figure 2). Within this approach, dentin bonding effectiveness is primarily governed by micromechanical retention, especially through hybrid layer formation and resin tag penetration [30,36]. After phosphoric acid etching, adhesive monomers infiltrate the demineralized dentin matrix, creating a micrometer-scale hybrid layer [37]. Secondary interactions, such as van der Waals forces and hydrogen bonding, contribute minimally to adhesion, and the formation of stable chemical bonds with the exposed collagen matrix remains unlikely [36]. ZBU contains a ternary solvent system. The system contains a high percentage of ethanol (30–40%), water, and butanone, which provide a balanced combination of viscosity, surface tension, and vapor pressure. These physicochemical properties may promote effective diffusion of resin monomers into dentine. They also help avoid the limitations of highly volatile solvents, thereby enhancing the adhesive–dentine interaction [23,38]. Additionally, ZBU is a HEMA-free adhesive system with fluoride-releasing capability. The exclusion of HEMA reduces the intrinsic hydrophilicity of the adhesive, thereby limiting water sorption and decreasing susceptibility to hydrolytic degradation [3,39]. Furthermore, the absence of HEMA has been reported to promote the formation of a more stable and well-organized 10-MDP nano-layering at the adhesive surface, which contributes to enhanced bonding potential, particularly in SE mode [3,40].

In the present study, CUBQ exhibited bond strength comparable to that of CBU, despite being applied passively, whereas CBU was actively rubbed. According to the manufacturer, the performance of CUBQ is attributed to its reduced HEMA content and distinct hydrophilicity compared with conventional systems, potentially eliminating the need for active application or an extended waiting time [21,41]. Consistent with previous reports highlighting the importance of adhesive–substrate contact time and interaction with the smear layer, it can be inferred that sufficient chemical interaction can still be achieved under passive application [42,43]. The comparable performance between CUBQ and CBU may reflect the ability of CUBQ to achieve adequate monomer infiltration and interaction with dentin despite the absence of active agitation. CUBQ exhibited limited penetration into dentin in the ER mode, likely due to insufficient monomer diffusion into the collagen network under passive application. However, its bond strength is similar to that of CBU, suggesting that the adhesive performance is influenced not only by the penetration depth but also by the chemical composition. Notably, the hydrophilic amide monomers in CUBQ enhance dentin wettability, facilitating more uniform infiltration and stable chemical bonding. Long resin tags formed in ER mode have been reported not to significantly contribute to bond strength [44,45]. While some studies found that CUBQ exhibited bonding performance comparable to other universal adhesives [42,46], consistent with our findings, others reported lower bond strength values [38,42]. These differences can be attributed to variations in methodological design, such as smear layer formation, the test method, and the composite material used [21].

All adhesives in the ER mode demonstrated lower reliability than those in the SE mode. SE adhesives simultaneously demineralize dentin and infiltrate resin monomers, thereby minimizing the risk of collagen collapse and incomplete resin penetration. Moreover, the partial preservation of hydroxyapatite around collagen fibrils in the SE protocol enables functional monomers, such as 10-MDP, to establish chemical interactions with residual hydroxyapatite [3]. In contrast, in the ER protocol, excessive demineralization followed by insufficient resin infiltration may compromise bonding effectiveness [6]. Also, it has been reported that MMP release is greater in the ER protocol than in the SE protocol, leading to collagen degradation [47]. Previous studies reported higher bond strength for CUBQ and CBU in SE mode than in ER mode [48,49,50,51], whereas ZBU showed similar performance in both modes [38]. These differing results may be explained by methodological differences [21].

In the present study, thermocycling did not affect the bond strength of the tested adhesives, irrespective of the application mode. Previous studies have reported conflicting findings regarding the effect of thermocycling on bond strength [52,53,54], which have been mainly attributed to differences in adhesive composition, thermocycling protocols, and specimen design. The use of resin–dentin beams bonded to flat dentin surfaces may limit thermal stresses at the adhesive interface and reduce shrinkage- and temperature-induced stresses due to the low C-factor [53,55]. Accordingly, thermocycling applied to flat surfaces has been stated to have minimal influence on bond strength, whereas the same aging protocols applied to restored cavities result in a significant reduction in bonding performance [56].

Similarly, in the present study, the pH-cycling model had no significant influence on bond strength. The available literature on the effect of pH cycling on adhesive–dentin bond strength is limited; however, existing evidence suggests that its potential impact is associated with structural degradation and erosion at the adhesive interface, as well as mass loss of adhesives following exposure to acidic conditions [29,57]. Some studies have reported a significant reduction in bond strength after aging with pH cycling [29,58]. In contrast, other investigations have demonstrated that pH cycling does not adversely affect bond strength to dentin [59,60], which is consistent with the findings of the present study. These discrepancies among studies may be attributed to differences in adhesive systems and experimental designs. The presence of the functional 10-MDP monomer may have played a crucial role in preserving bond strength after both pH cycling and thermocycling, due to its chemical interaction with dentin and its potential to inhibit increased matrix metalloproteinase activity under acidic conditions [61,62,63]. Furthermore, acid–base challenges may have promoted the formation of an acid–base resistant zone, rendering them less susceptible to degradation [60,64].

The DC is a critical parameter influencing the polymerization efficiency of dental adhesives and, consequently, their bond strength [65,66]. However, the relationship between DC and bonding performance remains controversial, with previous studies reporting conflicting results [67,68]. In the present study, CBU and ZBU exhibited significantly higher DC values than CUBQ. It has been suggested that both functional monomers and photoinitiator systems play a key role in determining the DC of adhesive materials. In particular, incompatibility between hydrophobic photoinitiators and hydrophilic monomers may lead to phase separation, thereby reducing the overall DC [69]. The presence of a hydrophilic amide monomer together with the hydrophobic photoinitiator camphorquinone in CUBQ may explain its lower DC compared to CBU and ZBU [70].

Additionally, the solvent content of adhesive systems has been considered as a crucial factor influencing polymerization. Previous studies have shown that higher solvent concentrations prior to polymerization lead to lower degrees of conversion [71]. Specifically, the presence of water as a solvent in SE adhesive systems has been reported to decrease DC and adversely affect their mechanical properties [72,73]. The findings of the present study suggest that the solvent system of CUBQ may be less effective in solvent evaporation than that of ZBU and CBU, leading to increased water retention within the polymer matrix and, consequently, lower DC values. Although a previous study reported higher DC values for CUBQ [74], this discrepancy may be due to methodological differences, as DC was measured after adhesive application to dentin. Additionally, thermocycling-induced post-polymerization and interactions with hydroxyapatite have been reported to increase DC [75], which may explain the lower DC observed for CUBQ in the present study.

This study has several limitations. First, it was performed under in vitro conditions, and the findings may not fully replicate clinical variables such as oral humidity, pH fluctuations, mechanical stresses, and biological interactions, all of which can influence adhesive performance [76]. Second, the study involved 10,000 thermal cycles, corresponding to approximately one year of clinical period, which may be considered a relatively short period for predicting long-term outcomes. Additionally, factors such as cavity configuration (C-factor) and occlusal loading were not considered. Therefore, long-term clinical studies are necessary to validate these results and to better understand the durability and performance of these adhesives under real-life conditions.

## 5. Conclusions

Within the limitations of this in vitro study, ZBU demonstrated the highest overall bond strength performance across all etching modes, which may be attributed to its favorable interfacial interactions with dentin. Following aging, no significant differences were observed among the adhesives, indicating that the aging protocols did not significantly influence bonding performance under the tested conditions. Although aging had no significant effect on bond strength, adhesives applied using the SE protocol exhibited greater reliability than those applied using the ER protocol, as indicated by Weibull analysis. Nevertheless, further long-term clinical and in vivo studies are required to confirm these findings and clarify their clinical significance.

## Figures and Tables

**Table 1:** Adhesive composition and recommended application methods.

Adhesive (Manufacturer)	Chemical Components	Application Instructions According to Manufacturers
Clearfil S3 Bond Universal (Kuraray Noritake Dental, Tokyo, Japan) Lot: 690062	Bis-GMA, HEMA, 10-MDP, hydrophilic aliphatic dimethacrylate, camphorquinone, colloidal silica, accelerators, silane, ethanol, initiators, water Mild (pH = 2.3)	Apply to the dentin surface using a microbrush, actively rubbed for 10 s, gently air-dried for 5 s, and polymerized for 10 s.
Clearfil Universal Bond Quick (Kuraray Noritake Dental, Tokyo, Japan) Lot: AS0431	Bis-GMA, 10-MDP, HEMA, hydrophilic amide monomers, colloidal silica, silane coupling agent, ethanol, camphorquinone, sodium fluoride, water Mild (pH = 2.3)	Apply to the dentin surface using a microbrush, gently air-dried for more than 5 s, and polymerized for 10 s.
Zipbond Universal (SDI, Victoria, Australia) Lot: 1233262	UDMA, 10-MDP, aliphatic dimethacrylate, hydrophilic aliphatic monomethacrylate, acidic monomethacrylate, initiator, ethanol, butanone, fluoride, water Mild (pH = 2.5)	Apply to the dentin surface by rubbing with a microbrush for 10 s, left on the dentin surface for 10 s, gently air-dried for more than 5 s, and polymerized for 10 s.

Bis-GMA: Bisphenol A-glycidyl methacrylate; HEMA: Hydroxyethyl methacrylate; 10-MDP: 10-Methacryloyloxydecyl dihydrogen phosphate; UDMA: Urethane dimethacrylate resin.

**Table 2:** Mean microtensile bond strengths and standard deviations (± SD) for adhesives in different etching modes after different aging protocols.

Adhesive	Application Mode	24 h	Thermocycling	pH-Cycling
CUBQ	SE	26.39 ± 7.97 ^AB^	33.84 ± 5.94 ^A^	30.91 ± 11.58 ^A^
	ER	20.84 ± 11.52 ^A^	25.97 ± 12.85 ^A^	27.60 ± 5.45 ^A^
CBU	SE	26.64 ± 6.60 ^AB^	31.36 ± 9.81 ^A^	29.55 ± 7.73 ^A^
	ER	25.51 ± 10.15 ^AB^	25.15 ± 15.04 ^A^	25.17 ± 12.92 ^A^
ZBU	SE	33.83 ± 9.92 ^AB^	39.48 ± 14.32 ^A^	33.76 ± 6.22 ^A^
	ER	36.94 ± 14.71 ^B^	32.24 ± 11.51 ^A^	27.82 ± 8.22 ^A^

Different uppercase letters in the vertical direction indicate a significant difference (*p* < 0.05).

**Table 3:** Comparison of conversion degree values (±SD) of the adhesives.

Adhesives	Min-Max	Mean	*p* Value
CUBQ	32.40–53.00	42.60 ± 8.81 ^A^	0.012
CBU	45.00–94.70	65.62 ±18.05 ^B^	
ZBU	63.70–73.80	66.54 ± 4.20 ^B^	

Different letters indicate significant differences (*p* < 0.05).

## Data Availability

The raw data supporting the conclusions of this article will be made available by the authors on request.
